# Heterogeneity of Cholangiocarcinoma Immune Biology

**DOI:** 10.3390/cells12060846

**Published:** 2023-03-08

**Authors:** Francesca Vita, Irene Olaizola, Francesco Amato, Colin Rae, Sergi Marco, Jesus M. Banales, Chiara Braconi

**Affiliations:** 1 School of Cancer Sciences, University of Glasgow, Glasgow G61 1QH, UK; francesca.vita@unito.it (F.V.); 2496589a@student.gla.ac.uk (F.A.); colin.rae@glasgow.ac.uk (C.R.); sergi.marco@glasgow.ac.uk (S.M.); 2 Department of Oncology, University of Turin, 10043 Turin, Italy; 3 Department of Liver and Gastrointestinal Diseases, Biodonostia Health Research Institute–Donostia University Hospital, University of the Basque Country (UPV/EHU), 20014 San Sebastian, Spain; irene.olaizola@biodonostia.org (I.O.); jesus.banales@biodonostia.org (J.M.B.); 4 IKERBASQUE, Basque Foundation for Science, 48009 Bilbao, Spain; 5 National Institute for the Study of Liver and Gastrointestinal Diseases (CIBERehd, “Instituto de Salud Carlos III”), 28029 Madrid, Spain; 6 Department of Biochemistry and Genetics, School of Sciences, University of Navarra, 31008 Pamplona, Spain; 7 Beatson West of Scotland Cancer Centre, Glasgow G12 0YN, UK

**Keywords:** cholangiocarcinoma, liver, immune cells, tumor microenvironment, heterogeneity, cancer-associated fibroblasts, cancer stem cells

## Abstract

Cholangiocarcinomas (CCAs) are aggressive tumors arising along the biliary tract epithelium, whose incidence and mortality are increasing. CCAs are highly desmoplastic cancers characterized by a dense tumor microenvironment (TME), in which each single component plays a fundamental role in shaping CCA initiation, progression and resistance to therapies. The crosstalk between cancer cells and TME can affect the recruitment, infiltration and differentiation of immune cells. According to the stage of the disease and to intra- and inter-patient heterogeneity, TME may contribute to either protumoral or antitumoral activities. Therefore, a better understanding of the effect of each immune cell subtype may open the path to new personalized immune therapeutic strategies for the management of CCA. In this review, we describe the role of immune cells in CCA initiation and progression, and their crosstalk with both cancer-associated fibroblasts (CAFs) and the cancer-stem-cell-like (CSC) niche.

## 1. Introduction 

Cholangiocarcinoma (CCA) comprises a heterogeneous group of malignancies arising along the biliary tree epithelium [1]. According to their anatomical origin, CCAs are classified as intrahepatic (iCCA), perihilar (pCCA) or distal CCA (dCCA), which share similarities but also have important inter-tumor and intra-tumor differences that can affect the pathogenesis and outcome [2,3]. The global trend of CCAs indicates an increase in their incidence (0.3–6 per 100.000 inhabitants per year) over the past few decades, and they now account for ~3% of all gastrointestinal cancers [2,4,5]. Patients with CCA are frequently asymptomatic in early tumor stages, thus, most of them (~70%) are diagnosed at advanced phases when the disease is widespread. Late diagnosis, together with the high chemoresistant nature of these tumors, compromises the possible curative therapeutic options and contributes to their dismal prognosis [2,3]. Currently, surgical resection or liver transplantation are the only potentially curative options for patients with CCA, although they are indicated in less than 30% of patients and chances of cancer recurrence are high [6]. For unresectable cases, palliative treatment, comprising the combination of gemcitabine (Gem), cisplatin (CisPt) and immune checkpoint inhibitors (ICI) remains the only possible option providing a median overall survival of 12 months [3]. Nevertheless, as an exception, CCA patients with microsatellite instability (MSI), which are characterized by an increased presentation of tumoral antigens, show a good response to treatment with ICI [7,8].

CCAs are highly desmoplastic tumors presenting a very dense tumor microenvironment (TME). Even though tumor epithelial cells (i.e., cholangiocytes) are generally considered the coordinators of tumor growth, the crosstalk between the tumor and its stroma is known to play a remarkable role [9,10,11,12,13]. The CCA stroma consists of a complex network of extracellular matrix proteins and diverse cell types, including infiltrating immune cells (e.g., macrophages, neutrophils, natural killer or T cells), endothelial cells, mesenchymal stem cells and cancer-associated fibroblasts (CAFs) which interact with the tumor epithelium to support and sustain the different defining features of cancer cells [14]. Despite the crosstalk between tumor and stroma being thought to drive cancer progression and metastasis, the exact mechanism by which the TME acts is multifaceted and only partially understood.

In recent years, the understanding of the immune landscape and the design of immune-targeted therapies has taken a significant role in treating cancer. In this review, we describe the role of the innate immune system, including monocytes, macrophages, myeloid-derived suppressor cells, neutrophils and natural killers, as well as the adaptive immune system, including B lymphocytes and T lymphocytes, in CCA initiation and progression. Their crosstalk with both CAFs and cancer stem cells will be discussed, highlighting their relevance as possible therapeutic targets in CCA.

### Immune System

The immune system comprises a tiny-regulated system of biological reactions that aim at defending the body from pathological conditions. It is made up of the innate immune system and the adaptive immune system; the former being responsible for a non-specific response and the latter for a more specific one. Upon encountering foreign pathogens or conditions, the innate immune system becomes activated. Therefore, it recognizes the presence of immunostimulant molecules on the pathogens (pathogen-associated molecular patterns, PAMPs) and/or on damaged cells (damage-associated molecular patterns, DAMPs) and triggers a nonspecific response. However, when the innate immune system is not able to remove the foreign substances, the adaptive immune system becomes activated and an antigen-mediated response is initiated. The collaboration between those two systems is fundamental to defend the body from harmful agents, such as cancer cells.

The liver is an organ characterized by its robust and unique immunological features including the presence of a predominant innate immune system due to exposure to intestinal bacteria and toxins [15]. However, the liver must also remain alert to potential harmful pathogens such as infectious agents or cancer cells [16]. Tumor cells might use the tolerogenic features of the liver to promote immune tolerance, and thus enhance tumor progression, which has a direct impact on patients’ outcomes. In this context, unraveling the immune response to CCA is crucial in order to develop effective immunotherapeutic strategies.

## 2. Innate Immune System

### 2.1. Monocytes

**Figure 1:**
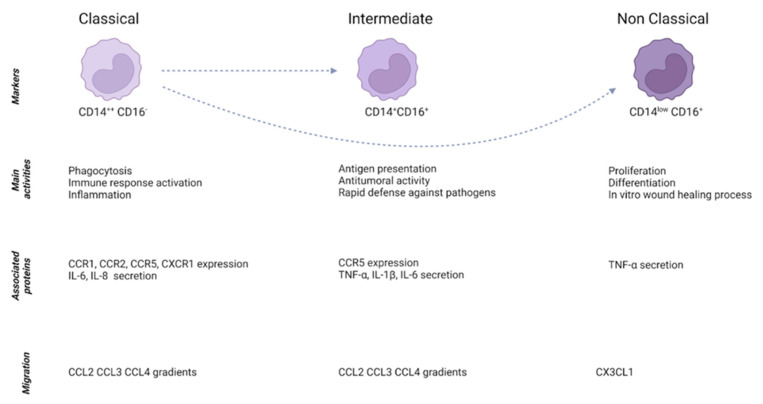
Traditional classification of monocytes. Traditionally, monocytes are classified as classical, intermediate and non-classical. Intermediate and non-classical monocytes can differentiate from the classical subset. The figure shows markers, molecules and activities of each subset.

Monocytes are plastic blood cells that are mobilized from the circulation to both healthy and pathological sites where they play a fundamental role in supporting tissue homeostasis, in initiating and propagating immune responses as well as in ending them [17]. Monocytes have been traditionally classified as classical (CD14^++^CD16^−^) and non-classical (CD14^low^CD16^+^) [18]. Nonetheless, a population categorized as “intermediate” (CD14^+^CD16^+^ HLA-DR^+^CD86^+^CD11C^+^) has also been described [19]. Of those three subsets, classical monocytes have been suggested to be the source of differentiation for non-classical and intermediate monocytes [18]. Classical monocytes exhibit high expression levels of genes involved in the regulation of phagocytosis and immune response and they secrete pro-inflammatory molecules, whereas non-classical monocytes are associated with proliferation, differentiation, wound healing processes and tumor necrosis factor (TNF)-α secretion [18]. Intermediate monocytes have been described as the population with the strongest antitumoral activity and highest expression of molecules involved in antigen presentation [20] (Figure 1). However, the specific function of each subset has not been elucidated yet, and several findings have shown overlapping functions. More recently, studies based on single-cell RNA analysis demonstrated the presence of several subsets of monocytes in the liver, highlighting the huge heterogeneity of immune cells in this organ and the potential presence of subgroups with different functions [21].

In cancer, monocytes are key regulators of tumorigenesis and tumor progression [22]. According to their strong heterogeneous characteristics, they can exert different functions resulting in either promoting or hindering tumor growth and metastatic spread [22]. This heterogeneity is dependent on the crosstalk with the TME, as well as on the cancer type and the stage of tumor growth [22].

Monocytes are mobilized to cancer tissue and metastatic sites during every step of cancer progression [23]. Once they reach the tumor bed, they can exert their antitumor activity by preventing metastatic spread [24,25], inhibiting regulatory T cells (Tregs) and releasing immune-modulatory cytokines, such as interferon (IFN) or TNF [26]. It is noteworthy, that this action can be suppressed by tumor cells through interleukin (IL)-8 release. Conversely, monocytes can also enhance tumor growth by differentiating into tumor-associated macrophages (TAMs) [27], suppressing T cell function [27,28] or recruiting T regs [29], and thus promoting angiogenesis [30], metastatic spread and extracellular matrix (ECM) remodeling [31].

**Figure 2:**
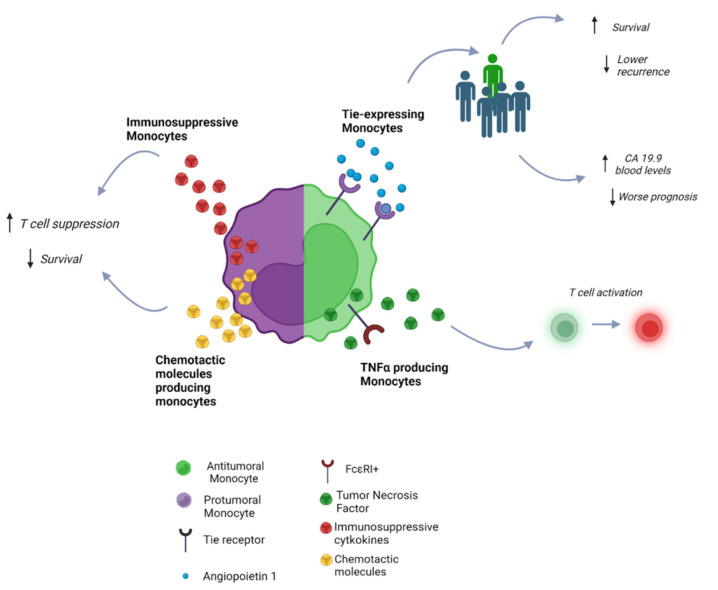
Monocyte subclasses in CCA. Tie-expressing monocytes and TNF-α-producing monocytes are identified as subgroups of “antitumoral monocytes” (right, green) in CCA, whereas immunosuppressive monocytes and chemotactic-molecule-producing monocytes are classified as subsets of “protumoral monocytes” (left, purple).

The crosstalk between monocytes and cancer cells in CCA has been demonstrated to modulate the role that monocytes may play in CCA progression, being responsible for both their protumoral and antitumoral function. In this regard, several monocyte subclasses are present in CCA and each has a distinct activity (Figure 2). In particular, a subset of monocytes expressing Tie has been shown to counteract tumor growth. Tie is a receptor tyrosine kinase (RTK) capable of binding angiopoietins (Ang) 1 and 2, which have been identified as negative and positive promoters of angiogenesis, respectively, in cancer. High Ang-1 expression and high infiltration of Ang-receptor bearing Tie-expressing monocytes (TEM) in a subgroup of pCCA patients exerted a protective role, resulting in lower tumor recurrence and increased survival [24]. In iCCA, lower TEM infiltration is associated with higher blood levels of CA19.9, which is associated with disseminated disease [25].

New findings have pointed to an antitumoral role of a subset of monocytes expressing the TNF-α producing high affinity receptor (FcεRI^+^), which mediates inflammation and T-cell activation. FcεRI^+^ monocytes were found to be lower in patients with CCA compared to healthy controls [26].

On the contrary, when secreting high levels of immunosuppressive cytokines and chemotactic molecules, monocytes can both directly mediate CD4^+^ T-cell suppression and induce the expression of SOC3, which renders T cells functionally unresponsive. These characteristics correlate with a worse prognosis and has been associated with resistance to anti-programmed-cell-death (PD)-1 treatment in patients with CCA [28]. 

Overall, the intimate relation between monocytes and cancer cells suggests that monocytes may be promising targets for future therapies against CCA. Nevertheless, only few findings have identified new subgroups of monocytes involved in CCA progression. Hence, a deeper understanding of the heterogeneity of this population of immune cells is required to clarify the differences between cancer patients and healthy individuals.

### 2.2. Tumor-Associated Macrophages

The “scavengers” of the body are remarkably characterized by phenotypic heterogeneity and functional diversity. Their activity can vary significantly, ranging from anti-inflammatory to pro-inflammatory, from immunogenic response to immune tolerance and from mediating tissue destruction to regulating tissue repair [32]. Macrophages are involved in both innate and adaptive immunity, where they exert different functions including phagocytosis, antigen presentation, defense against microbial cytotoxicity and secretion of cytokines [32]. Once macrophages become active, they can polarize into M1 (classically activated) or M2 (alternatively activated) [33]. M1 macrophages are activated by lipopolysaccharide (LPS), they express CD80 and CD86 and they are involved in pro-inflammatory responses [34]. On the contrary, M2 macrophages are activated by IL-4 and IL-10, they express higher levels of CD163 and CD206, they are involved in anti-inflammatory responses and repair and are generally considered immunosuppressive [34]. In any case, this traditional division appears to be oversimplified and several studies have shown the presence of more subsets.

Tumor-associated macrophages (TAMs) (CD68^+^ cells) are highly present in several cancer types, including CCA, where they infiltrate the tumor bed and its margins and correlate with poor prognosis [35]. Currently, some studies have shown that TAMs are in constant transition between the M1 and M2 phenotypes in tumor tissue, and the ratio of every subgroup is strongly regulated by the TME. According to these findings, in CCA, macrophages tend to polarize to the M1 phenotype at early stages of infection with Clonorchis sinensis and to the M2 phenotype during late stages of cholangiocarcinogenesis, contributing to fibrosis and bile duct remodeling [36]. Interestingly, when the M1 subset is predominant, CCA cells can express elevated levels of CD47, which act as a “*don’t eat me*” signal, blocking phagocytosis and favoring cancer [37].

Although they differentiate mostly from peripheral monocytes, some subgroups of TAMs can originate even from embryonic-derived resident macrophages and myeloid--derived suppressor cells (MDSC), under the stimulation of several molecules [35]. In this regard, macrophage colony stimulating factor (M-CSF) and granulocyte macrophage colony stimulating factor (GM-CSF) can orchestrate TAM differentiation via the NF-kB pathway, under the presence of TNF-like weak inducer of apoptosis (TWEAK)/fibroblast growth factor-inducible 14 (Fn14), which is upregulated in CCA [35]. Differentiation into TAMs can also be driven by CCA CSCs via IL-13, IL-34, and osteoactivin (OA) production [38]. 

Once differentiated, TAMs can express protumoral molecules and activate several pathways, hence, promoting proliferation, epithelial–mesenchymal transition (EMT), invasion, metastasis, angiogenesis and resistance to conventional therapies [39]. For instance, it has been demonstrated that WNT7B-expressing TAMs in CCA can drive cancer progression by stimulating the canonical *WNT* pathway and, thus, the transcription of genes involved in several protumoral processes, such as cell cycle transition [40]. During iCCA progression, M2 macrophages can support EMT transition via AKT3/PRAS40 phosphorylation, the activation of STAT-3 and the secretion of several cytokines and chemokines, such as IL-6, TNF-α and transforming growth factor-β1 (TGF-β1) [41].

Additionally, TAMs can also modulate tumor angiogenesis and lymphangiogenesis. In fact, high infiltration of TAMs correlates with the detection of high levels of angiogenic factors in tumors, such as VEGF-A and epiregulin [42]. Interestingly, the presence of macrophages expressing CD80 at the margins of CCA bed has been associated with chemoresistance and relapse [43]. Apart from their direct protumoral features, TAMs can strongly support cancer growth by suppressing the immune response. For instance, programmed-cell-death-ligand (PD-L1) expression on TAMs has been demonstrated to be elevated in both mouse models and tumor tissues from patients with CCA, suggesting that TAMs may suppress cytotoxic-T-lymphocyte (CTL) activity through PD-1/PD-L1 interaction [42]. The upregulation of PD-L1 expression in TAMs can be orchestrated by miR-183-5p, a known cancer-related miRNA, via the PTEN/AKT/PD-L1 pathway, rendering it a potential predictive biomarker for immune checkpoint blockade (ICB) therapy [44]. Furthermore, TAMs can increase immune escape by releasing IL-10, which results in blocking the activity of antigen-presenting cells (APCs), dendritic cells (DCs), cytotoxic T lymphocytes (CTLs) and CD8^+^ T cells [42].

Since TAMs are key regulators of CCA genesis and progression, further understanding of their specific characteristics and roles in CCA is required to help developing novel therapies based on either their suppression or the repolarization into M1 subset, aiming at improving the prognosis of CCA patients.

### 2.3. Myeloid-Derived Suppressor Cells 

**Figure 3:**
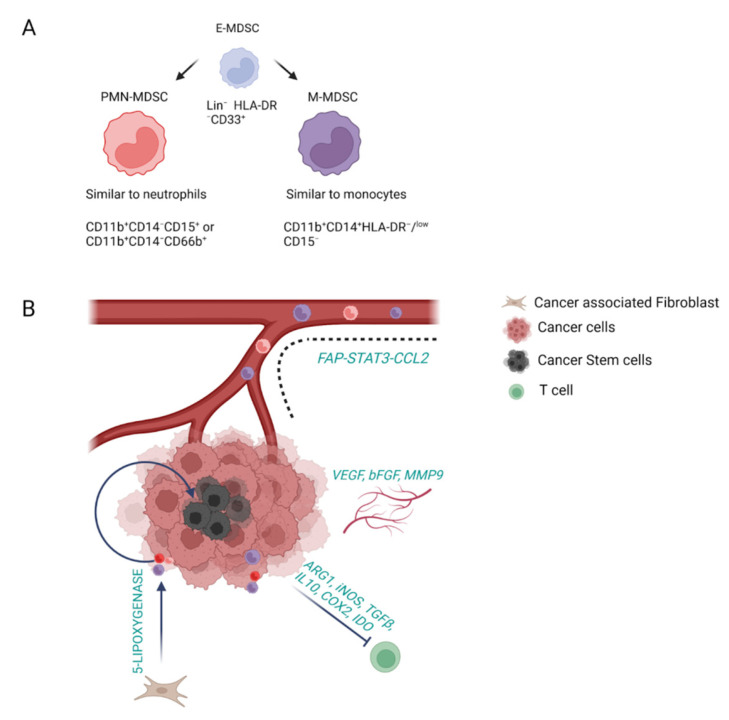
Classification, recruitment and functional activity of myeloid-derived suppressor cells (MDSC). (**A**) Subclassification of MDSC. MDSC are subgrouped into granulocitic (PMN-MDSC), representing the 80% of total MDSC, and monocytic (M-MDSC). (**B**) Recruitment and functional activity of MDSC. MDSC are recruited into CCA tissue mainly through the FAP–STAT3–CCL2 pathway and they cover their protumoral activity mainly by inhibiting T cells, by affecting angiogenesis and by sustaining the cancer stem cell niche.

As stated by their name, the main functional characteristic of myeloid-derived suppressor cells (MDSCs) is their immune-suppressive activity. Hence, MDSCs are present only in pathological states, such as chronic inflammation, autoimmune diseases and cancer [45]. It is noteworthy, that a strong correlation has been observed between MDSC levels, prognosis of patients with cancer, and response to chemotherapeutic agents such as cisplatin. Traditionally, MDSCs are subgrouped into granulocytic/polymorphonuclear (PMN-MDSCs), which are described as CD11b^+^CD14^−^CD15^+^ or CD11b^+^CD14^−^CD66b^+^, thus phenotypically similar to neutrophils; and monocytic (M-MDSC), defined as CD11b^+^CD14^+^HLA-DR^−^/^low^ CD15^−^ and, consequently, more similar to monocytes [45,46]. Among those subsets, PMN-MDSCs are the most abundant population, accounting for 80% of all MDSCs. In addition, a small population of cells among the early-stage MDSC (e-MDSC) (3% of total monocytes), which are defined as Lin^−^ (including lack of expression of CD3, CD14, CD15, CD19 and CD56) HLA-DR^−^CD33^+^, have been described to act as progenitors [45,46] (Figure 3A).

MDSCs differentiate through a multistep process: First, in pathological conditions, under the stimulation of STAT3, IRF8, C/EBP-β, Notch, adenosine receptor A2b signaling and NLRP3 [47], immature myeloid cells (IMCs) undergo expansion and inhibition of their terminal differentiation. Upon this, they are pathologically activated and differentiate into MDSC, which can be found not only in tumor tissue but also in spleen, bone marrow, blood and lymph nodes [47]. This whole process is strongly orchestrated by the tumor stroma which releases pro-inflammatory cytokines, enabling the signaling through the NF-κB pathway, STAT1, STAT6, prostaglandin E2 (PGE2) and cyclooxygenase 2 (COX2) [47]. Once differentiated, MDSCs reach the tumor through the guide of chemoattractive molecules, such as CXCL5 and CXCL2 [47]. CAFs can also guide MDSCs to CCA through the activation of the FAP-STAT3 pathway and CCL2 [47]. MDSCs exert their function by promoting tumor progression, angiogenesis, resistance to therapies and metastatic spread [45] (Figure 3B). Interestingly, these mechanisms of action are dependent on MDSC subgroups, as well as on the stage of the disease and the cancer type. It is worth mentioning that PMN-MDSCs have been shown to guide primary sclerosing cholangitis (PSC) into CCA [48], highlighting their role not only in cancer progression but also in the first steps of its initiation. The protumoral features of MDSCs are mainly covered by inhibiting T cells through arginase (ARG1), iNOS, TGF-β, IL10, COX2 and indoleamine 2,3-dioxygenase (IDO) release [45]. MDSCs can also guide tumor progression by remodeling the TME. Indeed, they can affect angiogenesis, through the release of VEGF, bFGF and MMP9, and support tumor recurrence and resistance to therapies by stimulating CSCs in iCCA [47]. This last process is mediated by CAFs, which shape MDSC function through the release of 5-lipoxygenase [49]. The reciprocal crosstalk between CAFs and MDSCs in cancer is fundamental, as they both modulate one another’s activity. In this regard, a recent work demonstrated that MDSC depletion in CCA models blocked the protumoral effect of CAF [50]. In addition, depletion of PMN-MDSC showed the possibility of restoring the response to anti-PD-1 therapies in murine models of CCA [51].

In conclusion, several findings have described the protumoral role of MDSCs in shaping CCA initiation, progression and resistance to therapies. It is noteworthy, that the depletion of MDSC in preclinical studies resulted in a better response to anticancer treatments such as ICI; thus, therapies based on inhibiting MDSCs in CCA hold great promise for the future.

### 2.4. Neutrophils

Neutrophils have a central role in host defense and clearance of invading pathogens such as bacteria or fungi. However, little is known about their role in human cancer development. Neutrophils represent 50–70% of circulating leukocytes, thus being the predominant circulating leukocyte population [52]. In order to fulfill their well-established host defense function, neutrophils have to leave the circulation and enter the target tissue. There, they release several activating cytokines including TNF-α, IL-1 and IFN, which contribute to the engulfing and killing of invading microorganisms [53].

It is becoming increasingly clear that tumor-associated neutrophils (TAN) play an important role in cancer biology [52,53]. TAN seem to acquire different phenotypes in the tumor site, with the N1 phenotype being antitumorigenic and the N2 phenotype protumorigenic [16,54]. However, the exact mechanism responsible for the switch towards either the N1 or N2 phenotype has yet to be elucidated.

**Figure 4:**
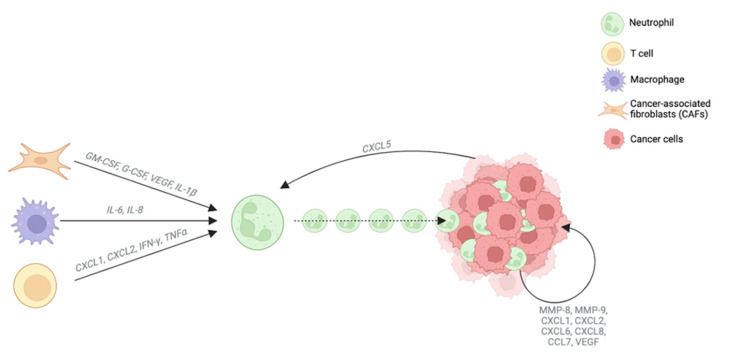
Neutrophil recruitment to the tumor site. TAN are recruited to the tumor site by interaction with different molecules produced by other cell types such as GM-CSF, granulocyte colony-stimulating factor (G-CSF), VEGF and IL-1β produced by CAFs; IL-6 and IL-8 produced by TAMs; and CXCL1, CXCL2, IFN-γ and TNF-α produced by T cells. Moreover, cancer cells also recruit neutrophils to the tumor site by releasing CXCL5. Once they reach the tumor site, TAN release different factors described to be highly involved in CCA biology such as MMP-8, MMP-9, CXCL1, CXCL2, CXCL6, CXCL8, CCL7 and VEGF.

There are few studies on the involvement of neutrophils in CCA pathogenesis and their function has not been well characterized yet. The crosstalk between TAN and the TME is well-established; indeed, TAN are sensitive to molecules produced by other cell types such as GM-CSF, granulocyte colony-stimulating factor (G-CSF), VEGF and IL-1β produced by CAFs; IL-6 and IL-8 produced by TAMs; and CXCL1, CXCL2, IFN-γ and TNF-α produced by T cells [54]. All these molecules promote neutrophil recruitment to the tumor. Likewise, cancer cells release CXCL5 which also enhances neutrophil recruitment through the activation of both the PI3K-Akt and ERK-1/2 signaling pathways [55]. Once they reach the tumor site, TAN release different factors such as MMP-8, MMP-9, CXCL1, CXCL2, CXCL6, CXCL8, CCL7 and VEGF, which have been described to be highly involved in CCA biology [9] (Figure 4).

Some studies have highlighted the role of TAN as potential novel biomarkers or therapeutic targets to treat CCA. On one hand, a positive correlation has been observed between high levels of intra-tumoral TAN and worse overall survival both in intrahepatic [56] and extrahepatic CCA patients [52,57]. Immunohistochemical and clinicopathological analyses revealed that patients with high CD15 expression, a marker of mature neutrophil distribution, in the TME had shorter disease-free survival times and worse overall survival [58]. Additionally, bile neutrophil gelatinase-associated lipocalin (NGAL) was suggested as a novel biomarker, alternative to CA19-9, to distinguish malignant pancreatobiliary cancers from benign biliary strictures [59]. On the other hand, TAN and TAM interaction has been shown to promote iCCA progression by activating the STAT3 signaling pathway, underlining the challenge in targeting one single component of the innate immune response [60].

The neutrophil-to-lymphocyte ratio (NLR) is emerging as a very promising prognostic value. A meta-analysis revealed that high NLR correlated with worse overall survival in CCA patients, suggesting its potential use as a prognostic biomarker of long-term outcomes [61]. In agreement with that, several studies have demonstrated that elevated NLR is associated with poor overall survival in patients with iCCA [62,63], and eCCA [64] undergoing surgical resection or first-line palliative chemotherapy [65]. Furthermore, data derived from the ABC-02 and BT-22 studies, not only confirmed the association between high NLR and worse overall survival and progression-free survival, but also suggested high NLR as a possible predictive marker of beneficial response to cisplatin and gemcitabine (CisGem) combination chemotherapy over gemcitabine alone in patients with advanced biliary tract cancer [66]. Similarly to NLR, serum-soluble programmed-death-ligand 1 (sPLD1) predicts survival in patients with advanced biliary tract cancer receiving palliative chemotherapy [67]. More recently the lymphocyte/monocyte ratio has shown a prognostic role in CCA patients undergoing chemotherapy, suggesting that tumor progression may be regulated by a fine balance between innate immune cells [68].

Furthermore, tumor infiltrating neutrophil (TINs) levels have recently gained importance as a predictor of beneficial response to adjuvant chemotherapy treatment in biliary cancer. Biliary cancer patients with low levels of TINs are more likely to respond to adjuvant chemotherapy and therefore have a reduced risk of compromised survival in comparison to those patients with high TIN levels [69]. In addition, TINs seem to inversely correlate with CD8^+^ T cells and positively correlate with Tregs. In this regard, the abundance of TINs and Tregs together with reduced CD8^+^ T-cell infiltrates is associated with poor prognosis in patients with CCA [57].

So far, the role of neutrophils in CCA progression has been under-investigated. Despite some studies showing very promising results from a prognostic and therapeutic point of view of CCA, further and deeper studies are required.

### 2.5. Natural Killer Cells 

Natural killer (NK) cells are a subset of effector lymphocytes from the innate immune system with potent cytolytic activity against microbial infection and tumor cells [70,71]. NK are “ready to kill” cells that rapidly recognize stressed cells such as cancer cells in an antigen-nonspecific way, thus, without prior sensitization. This recognition is mediated by the release of several cytokines and chemokines including perforin, proteases and granzymes [72]. Moreover, NK cells can activate TNF-family death receptors, such as Fas cell-surface death-receptor ligand (FasL) or TNF-related apoptosis-inducing ligand (TRAIL), directly killing cancer cells or cells that have been infected by viruses or bacteria [73,74]. Additionally, NK cells play an important role in cancer immunoediting. NK cells can secrete the pro-inflammatory cytokine INF-γ, mainly responsible for the induction of M1-like macrophage activation, promoting the defense against tumor surveillance [16,75]. Importantly, several preclinical and clinical studies have demonstrated an association between impaired NK cell function and NK cell deficiency and increased incidence of a variety of malignancies, including cancer [16,76].

Despite NK cells representing up to 30–40% of all liver lymphocytes [77] and being considered the largest immune cell subpopulation in CCA [78] little is known about their function in this type of cancer. The natural killer group 2 member D (NKG2D) receptor plays a fundamental role in NK cell-mediated clearance of tumor cells. Indeed, genetic alterations of the NKG2D receptor have been associated with impaired NK cytotoxic function, and thus with a higher risk of developing cancer [79]. Accordingly, patients with PSC harboring NKG2D-receptor polymorphisms have been shown to be more likely to develop CCA [79]. On the contrary, overexpression of NKG2D ligands correlates with improved disease-free and overall patient survival in CCA, hence, can be considered a good predictor of favorable prognoses [80]. Therefore, promoting the interaction of the NKG2D receptor and its ligand may hold great promise as a possible novel therapeutic strategy against CCA.

Since NK cells have an important cytotoxic activity, boosting NK cell recruitment to the tumor site or guaranteeing their correct function by repairing altered NK cells are important strategies that should be taken into consideration when developing immune-based therapies. For instance, high intra-tumoral expression of CXCL9, an IFN-γ-inducible chemokine, promotes NK cell recruitment to the tumor site and positively correlates with prolonged postoperative survival in patients with iCCA [81]. Moreover, a European study based on the DNA analysis of blood samples of patients with biliary cancer showed an imbalance in the genes encoding for NK immunoglobulin-like receptor (KIR) and human leukocyte antigen (HLA). These alterations may affect NK cell function and tumor surveillance, and, hence, should be considered when developing immune-based therapies [82]. As an example, in an ongoing phase I clinical trial for advanced/metastatic solid cancers, including CCA, nivolumab, a PD-1 inhibitor, is being tested in combination with the KIR inhibitor lirilumab. Although little is known about NK cell therapy for CCA from a clinical point of view, an in vivo study has demonstrated that the infusion of ex vivo-expanded human NK cells into CCA-xenograft mouse model halted tumor growth by means of NK cell-mediated cytolytic activity [83].

Nonetheless, the use of antibody-based therapies against different targets has also become a potential immunotherapeutic approach to tackle CCA. On one hand, the use of cetuximab, the monoclonal antibody against the epidermal growth factor receptor (EGFR) in an in vitro co-culture of human CCA cells and NK cells, significantly promoted CCA death by potentiating antibody-dependent NK cytotoxic activity [84]. Furthermore, a recent study has shown that major histocompatibility complex class I chain-related proteins A and B (MICA/B) drive NK cell antitumoral activity, providing preclinical evidence supporting MICA/B-specific 7C6 monoclonal antibody as a potential immunotherapeutic tool for iCCA [85]. Likewise, sensitizing CCA cells to immune cell cytotoxic activity with the aim of enhancing the immune system activation has also shown promising results and could be considered an alternative strategy. As an example, both cordycepin [86] and genistein [87] have been shown to effectively sensitize CCA cells to NK cytotoxicity, supporting their use as alternative immunomodulating agents in CCA. 

Overall, these studies provide clear insight into the different roles and clinical impacts of NK cells in CCA. Despite these findings holding promise, further preclinical studies are needed to investigate NK cell-based therapies in CCA. Aiming at enhancing NK cell cytotoxic activity, by both potentiating their recruitment to the tumor site as well as repairing abnormal NK cells, may prove to be a useful strategy to improve cancer-cell clearance.

### 2.6. Dendritic Cells 

Dendritic cells (DCs) are antigen presenting cells (APC) which are fundamental for T-cell activation, and subsequently for the activation of the adaptive immune system [71]. DCs are classified into classical DCs (cDCs) and plasmacytoid DCs (pDCs), the former considered highly phagocytic and the latter non-phagocytic [88]. cDCs recognize and interact with exogenous antigens in peripheral tissues, leading to adaptive immune response activation in secondary lymphoid organs via antigen presentation to CD4^+^ T cells and CD8^+^ T cells on MHC-II and MHC-I molecules, respectively [89]. Upon activation, pDCs release high levels of IFN-γ, required for antiviral immunity [16,90]. Moreover, DCs play an important role in promoting antitumor response. In fact, the lack of DCs, as well as the presence of DCs with abnormal activity, confers a poor prognosis. 

Although in the past few decades there has been an increased interest in better understanding the potential role of DCs in CCA, further attempts are required to elucidate their function. It is well known that patients with CCA have reduced levels of peripheral blood cDCs, as well as a significant decrease in TNF-α produced by cDCs, compared to healthy individuals [91]. Moreover, a correlation between CD83^+^ (mature) cDCs and CD4^+^/CD8^+^ T-cell infiltration at the invasive margin of the tumor has been observed in immunohistochemical analyses. In agreement with this, patients with a significantly higher number of mature CD83^+^ cDCs at the invasive margin had a lower incidence of lymph-node metastasis and overall better outcomes in comparison to patients with reduced levels of CD83^+^ cDCs [89]. By contrast, high levels of pDCs in peritumoral tissues of patients undergoing curative resection for iCCA are indicative of developing more aggressive tumors with advanced tumor-node-metastasis staging, more vascular/bile duct invasion and the presence of lymphatic metastases together with a higher risk of recurrence and poor overall survival [92].

As cDCs are associated with better patient outcomes, DC-based immunotherapies have been explored as potential therapeutic strategies in several preclinical and clinical models of CCA. An in vivo study using an orthotopic rat model of iCCA demonstrated that loading DCs with aspartate-β-hydroxylase (ASPH), a tumor-associated cell surface protein present in several tumors, induced not only suppression of tumor growth and metastasis, but also increased CD3^+^-lymphocyte infiltration levels into the tumors. This study highlights the possibilities of ASPH-loaded DCs as a novel therapeutic approach for iCCA and other hepatobiliary tumors [93]. Additionally, it is well described that the interaction between antigen-presenting cells and T cells is crucial for the initiation of the immune response. This interaction could be boosted by different strategies. The use of antibody-based therapies to tackle cancer has recently become a main research area. A recent study has demonstrated an association between low expression levels of CD40, a transmembrane protein necessary for APC activation, in CCA tissues and poor survival rates of patients. The combination of anti-PD-1 antibody and anti-CD40 agonistic antibody significantly reduced tumor growth compared to the IgG control, and to anti-PD-1 and anti-CD40 as monotherapies in subcutaneous, orthotopic and two plasmid-based murine iCCA models. Moreover, the combination of anti-CD40/PD-1 therapy led to an increased number of CD4^+^ and CD8^+^ T cells, CAF cells and myeloid cells in the tumor, which were shown to drive immunotherapy resistance in CCA [94]. These data are particularly relevant as anti-PD-1 drugs have entered the clinical management of biliary cancers. A phase I clinical trial of the anti-CD40 agonist (CDX-1140) alone or in combination with anti-PD-1 (pembrolizumab) is currently ongoing in solid cancers, including CCA [95]. On the other hand, in vitro activation of the immune checkpoint CD40/CD40L by endogenous expression of membrane-bound CD40L improves DC function and activates T-effector cells against several tumor types including CCA [96]. Currently, there are strategies being developed aimed at enhancing DC activation, such as ABL501, a bispecific antibody targeting both lymphocyte-activating gene 3 (LAG-3) and PD-L1, which in turn promotes effector CD4^+^ and CD8^+^ T-cell function. Importantly, increased levels of LAG-3^high^PD-1^high^ memory CD4^+^ T cells was observed by immune profiling analysis in the peripheral blood of patients with CCA relapse after treatment with Gem plus CisPt, showing a better response to ABL501. Based on this evidence, a first-in-human trial with ABL501 has been initiated [97].

Vaccines targeting DCs are another strategy to promote antitumor immunity. In recent years, a specific type of cellular immunotherapy based on transferring ex vivo-activated autologous effector T cells to patients has gained interest as a promising alternative approach for patients with CCA. Indeed, new strategies to improve the ex vivo activation of effector T cells are constantly arising aimed at upgrading their efficacy. Specifically, a recent study has developed self-differentiated monocyte-derived dendritic cells (SD-DC) by transducing isolated monocytes with lentivirus encoding granulocyte–macrophage colony-stimulating factor (GM-CSF) and interleukin-4 (IL-4), both of which are required for DC differentiation and activation. Moreover, the lentivirus also encoded for cAMP-dependent protein kinase type I-alpha regulatory subunit (PRKAR1A), which is an overexpressed protein that promotes T-cell activation, and subsequently their capacity to kill cancer cells. Therefore, these SD-DC expressing PRKAR1A boosted autologous effector-T-cell antitumor activity, significantly promoting their cytotoxic activity against CCA, thus representing a promising strategy to develop novel therapies to tackle CCA [98].

In conclusion, several strategies have been described with promising results mainly based on enhancing interaction of DCs and T cells and subsequently activating the immune system to defeat cancer. However, further understanding is required to delineate the exact role of DCs in cancer which could contribute to developing new and alternative therapeutic strategies. 

## 3. Adaptive Immune System 

### 3.1. T Lymphocytes

It is well known that T cells are a very heterogeneous group of cells involved in the adaptive immune response. Every subtype is characterized by different functional roles, and they are classified according to the T-cell-receptor (TCR) subunit and the core lineage marker [99]. In cancer, T cells are part of tertiary lymphoid structures (TLSs), a cluster of tumor-infiltrating lymphocytes (TILs), that can orchestrate antitumor immune responses [100]. High levels of CD8^+^ TILs positively correlate with good prognosis and response to immunotherapy [101], while infiltration of Tregs into the tumor area is associated with poor survival [102,103]. Tregs are a subset of T lymphocytes whose role it is to maintain peripheral tolerance by suppressing abnormal immune responses, thus preventing autoimmune disease [104]. Tregs are particularly active within the cancer tissue, where they support tumor growth and progression through their immunosuppressive features [104]. Additionally, it has been demonstrated that Tregs can modulate immunosuppression even from the periphery of the cancer tissue, through crosstalk with stroma and vasculature. Recent findings demonstrated the infiltration of Foxp3^+^ regulatory T cells into CCA tissue, where they support the tumor by inhibiting CD8^+^ TILs [103]. Further, Foxp3^+^ Treg/CD8^+^ TIL ratio (FCR) has been suggested as potential prognostic predictor marker of the disease. As several studies demonstrated, high FCR correlates with lymph-node metastasis and worse recurrence-free survival and OS in CCA [103]. Recently, new findings explored the role of other less-known subgroups of T cells, such as mucosal-associated invariant T (MAIT) cells, particularly present in the healthy liver, whose function is still controversial [105]. Interestingly, iCCA and pCCA tissues present lower levels of MAIT compared to healthy controls [106], supporting their potential antitumor activity. However, while the small amount of MAITs present in the tumor area was characterized by phenotypic and transcriptomic alterations, no changes were observed in the receptors involved in the interaction with tumor cells [106].

In cancer tissues, the activity of T cells is regulated by crosstalk with the TME which can shape them into a state of anergy, exhaustion, senescence and stemness [107]. Anergy is described as a hyporesponsive state of incomplete activation with low production of IL-2, which may be regulated by Erg2 [108]. Anergic T cells can induce tolerance in the periphery, contributing to tumor immune escape [108]. Exhausted T cells, under the stimulation of the B7-H1/PD-1 pathway, are characterized by low cytokine expression and poor effector function [107]. Additionally, they overexpress other inhibitory receptors, including T-cell immunoglobulin and mucin-domain-3 protein (Tim-3), Lag-3, cytotoxic T-lymphocyte antigen-4 (CTLA-4), and T-cell immunoglobulin and ITIM domain (TIGIT) [109,110], which can contribute to the loss of cytotoxic activity. In this regard, LAIR2 expression was found to be a significant marker of exhaustion of T cells in CCA [111]. Additionally, senescence in T lymphocytes is characterized by telomere shortening, loss of CD28 expression, cell cycle arrest, defective effector function and negative regulator activity [107]. Ultimately, T-cell stemness is described as the capacity of self-renewal and generation of memory T cells [107]. Apart from a few findings regarding exhaustion, T-cell responsive state in CCA has been under-investigated, thus, further studies are needed to better explore how the TME can shape their condition.

Immune-checkpoint inhibitors (ICI) are strategies targeting T cells, which aim at unblocking their cytotoxic activity when they are shaped to unresponsive states. In physiological conditions, immune checkpoints act as regulators of the immune response, thus preventing a strong response against healthy cells in the body. During cancer progression, tumor cells can inhibit T cells by expressing a wide range of surface molecules. Among them, the most famous interactions between cancer and T cells are the PD-1/PDL-1 axis, as well as CD80/CD86 with CD28/CTLA-4. Efficacy of ICI is positively affected by high tumor mutational burden (TMB), which is described as the number of mutations accumulated in the cancer genome. In this regard, CCA has been extensively demonstrated to be characterized by low TMB and, according to this, controversial results have been reported concerning ICI efficacy, suggesting that additional biomarkers of response are needed. In this regard, new findings recently showed that interferon signaling and major histocompatibility complex-associated genes are strongly involved in response to anti-PD-1 therapy in patients with hepatocellular carcinoma, suggesting that not only TMB affects ICI efficacy. In consequence, further studies confirming its validity even in CCA tissue, could open the path to stratify therapies according to the molecular markers expressed by each patient. Significantly, a phase III study recently demonstrated that the combination of durvalumab (anti PD-L1) plus standard chemotherapy reduced the risk of death by 20% compared to chemotherapy alone. As a result, the US Food and Drug Administration (FDA) has recently approved the use of durvalumab plus standard chemotherapy as first-line immunotherapy treatment in patients with CCA. 

Given their key role in the immune response, T-cell based therapies have been widely explored. In this context, adoptive cell therapy (ACT), which exploits the antitumor role of lymphocytes, has been shown to be more powerful than chemotherapy in hematological malignancies and some solid tumors. Historically, ACT can be based on both TIL and peripheral-blood-derived T cells [112]. After the isolation, lymphocytes are genetically engineered to express receptors enhancing their antitumor activity and then they are re-infused into the patient. For this purpose, lymphocytes can be engineered with TCR or with artificial receptors, such as chimeric antigen receptor (CAR). When engineered with CAR, T cells can specifically recognize tumor antigens and kill cancer cells. Clinical trials with CAR-engineered T cells recognizing glypican 3 (GPC3), mesothelin, and mucin 1 (MUC-1) are currently ongoing in patients with CCA. However, the challenge in making this strategy successful in CCA is the identification of a candidate gene which holds specificity for CCA cells. In addition, when engineered with TCR, T cells still need to link to MHC proteins, thus necessitating a functional immune system that can reach the site, and recognize and eliminate cancer cells. Furthermore, the very dense stroma characterizing CCA might hinder the efficacy of the therapy. Phase I/II clinical trials with TCR-based ACT are currently ongoing to determine their safety and efficacy against CCA.

In conclusion, several findings have supported the key role of T cells in fighting cancer, highlighting the potential role of cancer cells in inhibiting their cytotoxic features. Hence, restoring the intrinsic capability of T cells to killing is a rich promise in CCA. Nevertheless, the dense stroma that characterizes CCA might hinder ACD; thus, deeper preclinical and clinical studies are required to validate those strategies.

### 3.2. B Lymphocytes

In contrast to T lymphocytes, the role of B lymphocytes in CCA development and progression is still unclear. B cells represent the key regulatory cells of adaptive humoral immunity by producing antibodies against foreign substances [113]. Upon antigen recognition, naïve B cells bind to the antigens through the B cell receptors (BCRs) on their surface and become activated. Once activated, B cells differentiate into plasma cells which are responsible for antibody production [95]. However, B cells can also act as APC by presenting antigens to T cells, which then execute their cytotoxic activity. 

A subpopulation of B lymphocytes, known as regulatory B cells, are thought to promote carcinogenesis by inhibiting the antitumor immune response [114,115]. In contrast, tumor-infiltrating B lymphocytes (TIBs) have the capacity of limiting tumor growth by secreting immunoglobulins, enhancing the T cell response, and directly killing cancer cells, and therefore might improve a patient’s outcome [116]. Furthermore, B cells can also interact with helper T cells, altering tumor growth. The design of treatments that promote TIL presence in the tumors might represent an interesting approach to improve patients’ prognoses [117].

In CCA, B cells have been identified in tumor-infiltrating lymphocyte populations. Moreover, low-grade tumors present high levels of B lymphocytes, and this correlates with a favorable overall survival [111,118]. Furthermore, a recent single-cell study, aimed at elucidating the molecular characteristics of immune cells in iCCA, revealed that B cells were among the most abundant immune cells in the tumor tissue and in the peritumoral tissue, after T cells, NK cells and myeloid cells. This study demonstrated the presence of lower levels of B cells in the tumor tissue compared to adjacent peritumoral tissue [119]. However, given the limited number of studies on B cells in CCA, their relevance in this type of cancer as well as their feasibility as a potential immunotherapeutic target have not been elucidated yet. Thus, the role of B lymphocytes in CCA needs to be investigated in more detail.

## 4. The Crosstalk between Immune Cells and Cancer-Associated Fibroblasts

Cancer-associated fibroblasts (CAFs) are a heterogeneous group of activated fibroblasts with mesenchymal lineage origin. They are characterized by high expression of α-smooth muscle actin (α-SMA) and contribute to all steps of carcinogenesis, from neoplastic transformation to tumor progression [120]. Although their origin remains unclear and still controversial, CAFs seem to originate mainly from quiescent hepatic stellate cells (HSCs), tissue-resident portal fibroblasts and, to a lesser extent, from bone marrow-derived circulating mesenchymal stem cells [121,122,123]. It is still uncertain whether CAFs can originate from endothelial cells through endothelial-to-mesenchymal transition [124]. A recent single cell RNAseq analysis has described six subsets of CAFs in iCCA: vascular CAFs, matrix CAFs, inflammatory CAFs, antigen-presenting CAFs, EMT-like CAFs and lipofibroblasts. Each subtype of CAFs is characterized by a different gene expression profile and plays a different role in iCCA initiation and progression [125] (Table 1). Moreover, this study demonstrated that CD146^+^ vascular CAFs, which account for 57.6% of the total fibroblast population, secrete high levels of IL-6 which induces epigenetic alterations in iCCA cells, ultimately promoting their malignancy [125]. Likewise, another single-cell RNAseq study described the presence of two subpopulations of CAFs named inflammatory CAFs (iCAF) and myofibroblastic CAFs (myCAFs), the former being characterized by the expression of hyaluronan synthase 2 and the latter by the expression of HGF, thus directly promoting iCCA [126] (Table 1).

In cancer, CAFs promote new blood vessel formation and metastasis by enhancing immune-cell infiltration through the release of several cytokines and chemokines such as stromal cell-derived factor 1 (SDF-1) and IL-6, and by secreting several growth factors including endothelial growth factor, hepatocyte growth factor (HGF) and epidermal growth factor (EGF) [11,123]. Moreover, CAFs can express several matrix metalloproteases (MMPs) themselves or communicate with other components of the TME [127]. Migration behavior of cancer cells can also be activated by CAFs through the upregulation of integrin expression and cell-survival signaling pathways including the MEK–ERK and the PI3K–Akt signaling axes. Furthermore, CAFs contribute to the chemoresistant nature of tumors [128] by remodeling the extracellular matrix which acts as a physical barrier preventing chemotherapy from reaching tumor cells. Together, all these mediators contribute to boosting a pro-oncogenic environment, including that of CCA, supporting cancer-cell proliferation, angiogenesis, invasion and metastasis, thus, in general, tumor growth.

In CCAs’ TME, CAFs represent one of the most abundant cell types and are partly responsible for their dense stroma. Several studies that have focused on delineating the role of CAFs in CCA highlight a positive correlation between tumor growth and reduced survival with abundance of CAFs [129]. Indeed, a transcriptome profiling analysis revealed an association between high expression of several pro-inflammatory molecules in CCA stroma, such as IL6 and CXCR4, and the most malignant tumor phenotype [130]. CAFs can stimulate CCA growth through the release of short-range and direct morphogenetic signals such as Notch [131] or Sonic Hedgehog (SHH) [132], as well as via the secretion of several factors including HGF, PDGF-BB, HB-EGF and SDF-1 [123,133,134]. Particularly, once SDF-1 is released by CAFs, it binds to a receptor localized in the surface of CCA cells, CXCR4, and modulates the invasive capacity of CCA cells through the ERK1/2 and AKT pathways [133,135]. Moreover, a variety of MMPs (MMP1, MMP2, MMP9) [136] as well as CAF-secreted ECM components such as periostin [137] and tenascin-C [138], play a crucial role in tumor development [127,133]. However, CCA cells can also modulate the recruitment and activation of CAFs by the release of several factors including PDGF-D and TGF-β [139,140]. Malignant cholangiocyte-derived PDGF-D induces CAFs secretion of vascular growth factors (e.g., VEGF-A, VEGF-C) which attract lymphatic endothelial cells, hence, favoring CCA cell metastasis [141]. It is important to mention that not only CAFs can activate CCA cells and promote tumor development, but that also CCA cells can activate CAFs and, hence, restart the circle that ultimately leads to tumor progression.

Understanding the complex interplay between tumor cells, stroma, and both innate and adaptive immune cells infiltrating the tumor site may open a window for new therapeutic possibilities. It is well known that CAFs regulate the immune response to drive an immunosuppressive microenvironment, and that their crosstalk enhances tumor progression. CAFs can induce immunosuppression by producing TGF-β, which is a potent inhibitor of antitumor immunity, as it acts on NK cells, macrophages, neutrophils, CD8^+^ and CD4^+^ effector cells, and Tregs [122,123,142]. Likewise, thrombospondin-1, which is produced and released by CAFs [122], exerts immunosuppressive effects by TGF-β activation and direct interaction with immune cells [143,144]. Furthermore, fibroblast activation protein (FAP), which is also produced and released by CAFs, has also been described as an immune modulator [122]. Moreover, FAP has been shown to act as a persistent activator of fibroblastic STAT3, which subsequently leads to a CAF inflammatory phenotype.

Apart from inducing an immunosuppressive effect, CAFs, which are permanently active cells, can recruit immune cells from the bloodstream to the tumor site. For instance, FAP^+^ CAFs are the main cells responsible for CCL2 release, which enhances MDSC recruitment into the TME to exert their immunosuppressive function, and thus promote iCCA growth [50,133,145]. Another recent study showed that upon MDSC recruitment into the TME, CAFs might induce MDSCs to promote their stemness capacity via the 5-LO/LTB4-BLT2 signaling pathway. In vivo data showed that co-injection of CAFs and iCCA cells into the livers of nude mice, significantly enhanced the stemness of cancer. This effect could be counteracted by the elimination of CAFs or MDSCs [49,133].

**Figure 5:**
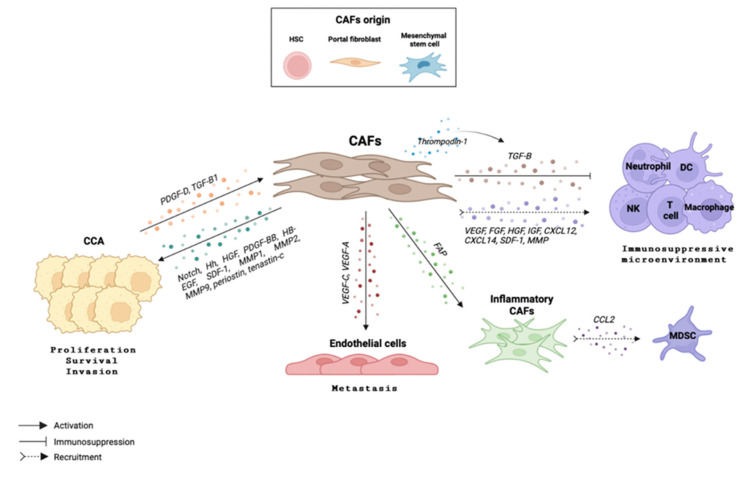
The crosstalk between immune cells and cancer-associated fibroblast (CAFs). CAFs are in continuous interaction with tumor cells and with other components of the tumor microenvironment, promoting tumor progression and metastasis. CAFs can directly induce CCA proliferation, survival and invasion through Notch, Hh, HGF, PDGF-BBB, HB-EGF, SDF-1, MMP1, MMP2, MMP9, periostin and tenascin-c release. Moreover, CAFs can activate endothelial cells via VEGF-C and VEGF-A release, and, hence, promote metastasis. Furthermore, CAFs can induce an immunosuppressive microenvironment by directly producing TGF-β, which is a potent inhibitor of antitumor immunity, as well as by releasing thrompodin-1, which subsequently activates TGF-β. Additionally, FAP^+^ CAFs, which are characterized by an inflammatory phenotype and are the main cells responsible for CCL2 release, enhance MDSC recruitment into the TME to exert their immunosuppressive function. Additionally, CAFs can also recruit immune cells from the bloodstream to the tumor site by releasing VEGF, FGF, HGF, IGF, CCL2, CXCL12, CXCL14, SDF-1 and MMP.

The recruitment of immune cells to tumor sites is strongly supported by CAFs through the release of several growth factors, as well as cytokines and chemokines (Figure 5). Among the released growth factors, the best-characterized ones are vascular endothelial growth factor (VEGF), fibroblast growth factor (FGF), hepatocyte growth factor (HGF) and insulin-like growth factor (IGF) [122]. Regarding cytokines and chemokines, CCL2, CXCL12 and CXCL14, as well as SDF-1 and IL-1 are prominent [127,128]. Moreover, co-culture studies demonstrated that the interaction between CAFs and CCA cells led to an increased production of IL-1 and CXCL5, by CAFs and CCA cells, respectively. Of note, CXCL5 is a well-described immune-cell chemoattractant through the activation of the PI3K–AKT and ERK1/2 pathways, as well as a well-known promoter of CCA migration and invasion [55,146]. Meanwhile, MMPs play a chemotactic role for leukocytes and modulate not only their proliferation but also their cytokine release [147]. Moreover, an association between CAF-mediated caveolin-1 (CAV1) and Foxp3^+^ TIL levels suggested that CAFs expressing CAV1 might recruit Tregs into the TME and subsequently mediate iCCA prognosis [148]. All these CAF-derived cellular mechanisms lead to the development of a pro-fibrotic and pro-angiogenic environment, which significantly promotes the initiation and progression of CCA [149].

In conclusion, in the past few decades, most research has focused on delineating the role of CAFs in tumor initiation and progression, including CCA. Their function has been well described and their fundamental role in tumorigenesis is undoubtable. However, despite great advances having been made, their interaction with cancer-associated immune cells, and in particular in CCA, still remains unclear. The interaction between CAFs and immune cells could represent a promising approach to develop new therapeutic strategies in the future, hence, further studies are needed in order to unravel this crosstalk.

## 5. The Crosstalk between Immune Cells and Cancer Stem Cells

Cancer stem cells (CSCs) are a subpopulation inside the tumor with self-renewal traits that exhibits characteristics of both stem cells and cancer cells [150]. CSCs are described as dormant chemoresistant cells that promote tumor relapse and metastasis, hence, they strongly affect patients’ outcomes, resulting in bad prognoses and short survival [151]. Further, they possess stemness-related genes and altered mitochondrial activity and they are characterized by high proliferation and epithelial–mesenchymal transition features. In CCA, it is hard to specifically identify the CSC population, as it is present as a subpopulation which shares not all the features characterizing CSCs. Consequently, according to their controversial nature, it is more suitable to refer to them as CCA CSC-like cells. CSC-like cells represent a significant part of the CCA tissue, accounting for 30% of the total mass [152] and they can express CD133, CD24, CD44, Sox2, CD49f, Sca-1 and CD117 [153]. These markers have been associated with in vitro and in vivo metastasis, relapse, resistance to therapies and invasiveness [153,154].

Generally, CSCs are strongly interconnected with the rest of tumor and with the other components of the TME (Figure 2). Of note, CSCs can release a wide range of molecules and act through several pathways to directly support cancer in every step of its progression, as well as to guide recruitment and shaping of immune cells [12]. Tumor cells are able to release growth factors and activate pathways themselves to assure CSC sustainment [12]. In this regard, the Wnt/β-catenin, Notch, Hedgehog, MAPK/ERK and TGF-β pathways have been demonstrated to be pathologically activated in CCA CSC-like cells [155]. All these pathways are associated with protumoral features and sustainment of CSCs [156]. In particular the Wnt/β-catenin pathway is associated with proliferation and apoptosis and its dysregulation can promote CSC self-renewal in early stages of carcinogenesis. Additionally, Notch signaling is implicated in iCCA progression [157]. The Hedgehog pathway is activated by stromal cells and it is required for proliferation of both hepatic stem cells and cancer cells, as well as migration and invasion [158].

**Figure 6:**
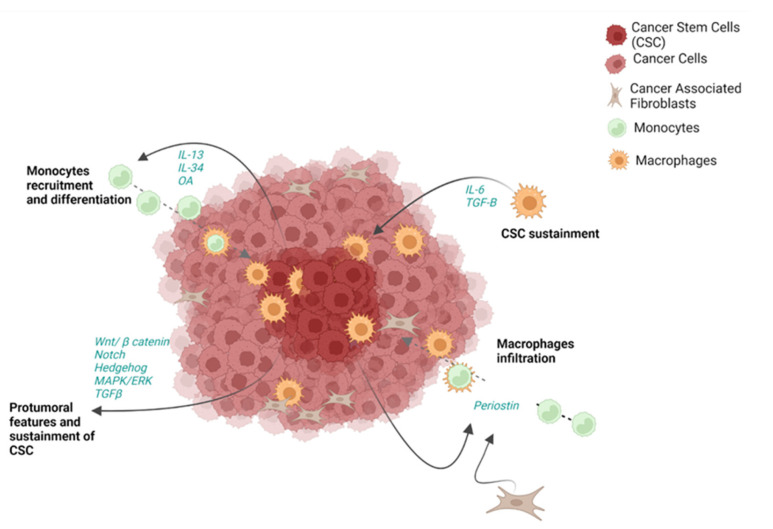
The crosstalk between immune cells and cancer stem cells. Cancer stem cells (CSCs) are involved in a circular network of connection with tumor cells and the other components of the tumor microenvironment (TME). Protumoral features and sustainment of CSC are supported by the dysregulation of Wnt/β, Notch, Hedgehog, MAPK/ERK and TGF-β which leads to proliferation, self-renewal and aggressiveness. *CSC sustainment* is also promoted by macrophages through the release of IL-6 and TGF-β. On the other hand, CSCs themselves can shape immune cell behavior, affecting monocyte recruitment and differentiation, by secreting IL-13, IL-34 and OA. Furthermore, periostin released by CSCs and CAFs can guide macrophage recruitment and infiltration in CCA tissue.

Although the crosstalk between CSCs and the TME has been described in several solid cancers, little is known about the contribution of CSC-like cells to CCA. Nevertheless, some studies showed the colocalization of CD163^+^ TAMs and CSC-like cell-related markers (CD44 or EPCAM) [38,42]; and the infiltration of macrophages in CCA seems to be guided by CSC-like cells and CAF through the release of periostin [38,159] (Figure 6). However, TAMs have also been shown to sustain CSC-like cells by releasing soluble mediators, including IL-6 and TGF-β [160], confirming a “circular support” of the two players. Furthermore, in vitro findings have demonstrated that CCA spheres can strongly attract monocytes through IL-13, osteoactivin (OA) and IL-34 release, inducing their differentiation to macrophages [38] (Figure 6). These findings have been confirmed in clinical settings, where IL-13, IL-34 and OA have been found to be increased in the blood of patients with CCA. Similar to monocytes and macrophages, MDSC are also strongly involved in CSC-like cell sustainment. In this regard, it has been demonstrated that CAFs can act through the 5-LO/LTB4-BLT2 axis, which results in shaping MDSC to support stemness features in CCA (Figure 6).

Stemness features are also conferred during EMT, when cells also gain invasiveness and resistance properties [161]. Among the transcriptional factors involved in this process, ZEB1 has been identified as an inducer in iCCA [162]. ZEB1 regulates the expression of tissue growth factor (CTGF), hepatocyte growth factor (HGF) and interleukin 6 (IL-6) and has been associated with resistance to 5-FU treatment in vitro [163]. As mentioned, CSC-like cells represent a significant part of the total tumor mass in CCA. Currently, several therapies have been developed to target CD133, EpCAM and CD44 and their efficacy has been demonstrated, especially in HCC, where it resulted in lower tumor growth, invasiveness, migration and adhesion [164,165]. However, these strategies still need to be explored in CCA. Furthermore, some findings clarified the relationship between CSC-like cells and the TME, but they are mainly limited to myeloid lineages. Consequently, further preclinical, and clinical studies are needed to better clarify their crosstalk with the other components of TME.

## 6. Conclusions and Future Perspectives

CCAs are highly desmoplastic tumors with dense heterogenous TME (Table 2), which is actively implicated in the regulation of CCA initiation, progression and resistance to therapies, hence, impacting the prognosis and survival of patients. Although some subsets of immune cells have been traditionally classified as positive or negative regulators of tumor growth, it is now becoming increasingly accepted that the crosstalk with cancer cells is able to strongly affect their phenotype in vastly heterogeneous ways. As extensively demonstrated, both tumor and immune cells can act through different pathways to shape each other towards different outcomes. As the immune compartment represents a large part of CCA tissue and it covers a crucial role in tumor progression, new therapies have been developed to target it. Taking into account the intrinsic signature of each single component, those strategies aim at inhibiting their activity and controlling their differentiation or engineering them to enhance their antitumoral features. Importantly, several preclinical findings suggest the potential of targeting the immune compartment, and clinical trials are currently ongoing in patients with CCA to confirm their validity in clinical settings. However, further studies are required to better clarify the role of some subgroups of immune cells whose contribution to CCA growth has been poorly characterized, and to elucidate the molecular mechanisms orchestrating their function. Furthermore, future works should also focus on clarifying the associations between cancer etiologies, their mutational profiles and the composition and interactions of the TME in order to open the path to new specific therapeutic strategies to tackle CCA. 

## Figures and Tables

**Table 1:** Cancer-associated fibroblast (CAF) subtypes. Data extracted from a single cell RNAseq analysis carried out by Zhang et al., JHEP, 2020 [125]. This study described the presence of 6 different subsets of CAFs in iCCA, highlighting their different gene expression and function.

Name	Type of Markers	Expressed Genes	Main Function
Vascular CAFs (vCAFs)	Microvasculature and inflammatory signatures	CD146 (MCAM), MYH11, GJA4, RGS5, IL-6 and CCL8	Vascular development
Matrix CAFs (mCAFs)	Extracellular matrix signatures	Collagen (COL5A1, COL5A2, COL6A3), periostin (POSTN), FN1, LUM, DCN and VCAN	Extracellular matrix
Inflammatory CAFs (iCAFs)	Inflammatory signatures	FBLN1, IGF1, CXCL1, IGFBP6, SLPI, SAA1 and complement genes (G3 and G7)	Complement activation
Antigen-presenting CAFs (apCAFs)	Major histocompatibility complex II (MHC-II) signatures	CD74, HLA-DRA and HLA-DRB1	Antigen presentation
EMT-like CAFs (eCAFs)	Epithelium specific signatures	KRT19, KRT8 and SAA1	Epithelial- mesenchymal transition
Lipofibroblasts	Lipid metabolism signatures	APOA2, FABP1, FABP4 and FRZB	Lipid processing

**Table 2:** The main components of the tumor microenvironment in cholangiocarcinoma, its biological significance and prognosis correlation.

Site of Expression		Biomarker	Biological Significance	Prognostic Correlation
Innate immune system	Monocytes	Angiopoietin 1 [25]	Negative regulator of angiogenesis, binding Tie receptor	Reduced metastatic incidence in hilar CCA patients
		Tie [24,25]	Receptor-binding angiopoietin factors	Low infiltration of Tie-expressing monocytes has been correlated with lower survival and higher levels of CA 19-9
		FcεRI^+^ [26]	T-cell activation	Better prognosis
	Macrophages	CD68^+^/CD206^+^ [35]	Tumor-associated macrophage marker	Worse prognosis
		CD274^+^ [42]	Modulation of immune response	Worse prognosis
	Myeloid-derived suppressor cells	CD11b^+^CD14-CD15^+^ [45]	Marker of polymorphonuclear myeloid-derived suppressor cells	PMN-MDSC affects PSC progression into CCA
		CD11b^+^CD14^+^HLA-DR- [45]	Marker of monocytic myeloid-derived suppressor cells	MDSCs promote angiogenesis, metastatic spread and tumor recurrence
	Neutrophils	TANs [52,56,57]	Tumor-associated neutrophils that can acquire N1 (antitumorigenic) or N2 (protumorigenic) phenotype	Worse overall survival
		CD15^+^ [58]	Marker of mature neutrophils and neutrophil distribution in the tumor microenvironment	Shorter disease-free survival time and worse overall survival
		NGAL [59]	Neutrophil gelatinase-associated lipocalin. Initially found in activated neutrophils	Malignant pancreatobiliary cancers
		NLR [61,62,63,64]	Neutrophil–lymphocyte ratio	Worse overall survival
		(sPLD1) [67]	Immune-checkpoint inhibitor that binds to its receptor PD-1 expressed by T cells and other immune cells to regulate immune responses	Predicts survival in advanced BTC patients receiving palliative chemotherapy
		TINs [69]	Tumor-infiltrating neutrophils	Poor prognosis
	Natural killer	NKG2D receptor [79]	Activating receptor that is mostly expressed on cells of the cytotoxic arm of the immune system	PSC patients with NKG2D-receptor polymorphisms are more likely to develop CCA
		NKG2D ligands [80]	Ligands binding to NKG2D receptor	Improved disease-free and overall patient survival
	Dendritic cells	CD83^+^ cDCs [89]	Mature DCs	Better patient outcome
		pDCs [92]	Plasmacytoid DCs with non-phagocytic function	Higher risk of recurrence and poor overall survival
		CD40 [94]	Costimulatory protein found on antigen-presenting cells, required for their activation	Low CD40—poor survival rates
		FCGR1A [94]	High affinity immunoglobulin gamma Fc receptor I, which plays a crucial role in the immune response	Better overall survival
Adaptive immune system	B lymphocytes	[111,118]		Favorable overall survival
	T lymphocytes	CD4^+^ [101]	Marker of regulatory T cells	High infiltration is correlated with worse prognosis
		CD8^+^ [102]	Marker of cytotoxic T cells	High infiltration is correlated with better prognosis
		LAIR2 [111]	Marker of T-cell exhaustion	Worse prognosis
Tumor microenvironment	Cancer-associated fibroblasts	[129]		Reduced survival
		FAP^+^ [50,133,145]	CAF inflammatory phenotype	Promote iCCA growth
	Cancer stem cells	CD133^+^[166]	Marker of CCA stem cell niche	Metastasis and cancer recurrence
		CD24^+^ [167]	Marker of CCA stem cell niche	Metastasis and cancer progression
		CD44^+^ [168]	Marker of CCA stem cell niche	Metastasis, chemotherapy resistance
		EpCAM [169]	Marker of CCA stem cell niche	Epithelial–mesenchymal transition, poor prognosis
		SOX2 [170]	Marker of CCA stem cell niche	Tumor growth, invasion, metastatic spread, poor prognosis
		CD49-f [171]	Marker of CCA stem cell niche	Tumor growth, invasion, poor prognosis
		Sca-1 [172]	Marker of CCA stem cell niche	Tumor growth, poor prognosis
		CD117^+^ [153]	Marker of CCA stem cell niche	Tumor growth, poor prognosis
